# Ethics of health research with prisoners in Canada

**DOI:** 10.1186/s12910-017-0189-6

**Published:** 2017-04-27

**Authors:** Diego S. Silva, Flora I. Matheson, James V. Lavery

**Affiliations:** 1 Faculty of Health Sciences, Simon Fraser University, Blusson Hall, Room 11300, 8888 University Drive, Burnaby, B.C V5A 1S6 Canada; 2 Centre for Research on Inner City Health, St. Michael’s Hospital, Toronto, Canada; 3 Dalla Lana School of Public Health, University of Toronto, Toronto, Canada; 4 Institute for Clinical Evaluative Sciences, Toronto, Canada; 5 Hubert Department of Global Health, Rollins School of Public Health, and Center for Ethics, Emory University, Atlanta, USA

**Keywords:** Prisoners, Research ethics, Indigenous

## Abstract

**Background:**

Despite the growing recognition for the need to improve the health of prisoners in Canada and the need for health research, there has been little discussion of the ethical issues with regards to health research with prisoners in Canada. The purpose of this paper is to encourage a national conversation about what it means to conduct ethically sound health research with prisoners given the current realities of the Canadian system. Lessons from the Canadian system could presumably apply in other jurisdictions.

**Main text:**

Any discussion regarding research ethics with Canadian prisoners must begin by first taking into account the disproportionate number of Indigenous prisoners (e.g., 22–25% of prisoners are Indigenous, while representing approximately 3% of the general Canadian population) and the high proportion of prisoners suffering from mental illnesses (e.g., 45% of males and 69% of female inmates required mental health interventions while in custody). The main ethical challenges that researchers must navigate are (a) the power imbalances between them, the correctional services staff, and the prisoners, and the effects this has on obtaining voluntary consent to research; and (b), the various challenges associated to protecting the privacy and confidentiality of study participants who are prisoners. In order to solve these challenges, a first step would be to develop clear and transparent processes for ethical health research, which ought to be informed by multiple stakeholders, including prisoners, the correctional services staff, and researchers themselves.

**Conclusion:**

Stakeholder and community engagement ought to occur in Canada with regards to ethical health research with prisoners that should also include consultation with various parties, including prisoners, correctional services staff, and researchers. It is important that national and provincial research ethics organizations examine the sufficiency of existing research ethics guidance and, where there are gaps, to develop guidelines and help craft policy.

## Background

The *Safe Streets and Communities Act* passed by the Parliament of Canada in 2012 emphasized, among other things, mandatory minimum sentences for certain offenses and less judicial discretion for sentencing. Though controversial, the passage of the legislation raised the public and political currency of criminal justice issues in Canada. The 2013 Ontario’s coroner inquest and jury into the death of Ashley Smith, the young woman who was allowed to commit suicide as prison guards looked on, ruled her passing a homicide and re-ignited a public debate about the health and welfare of prisoners in Canada. One of the jury’s recommendations was that prisoners with mental illnesses participate in “… planning, research, training and policy development…” regarding the provision of mental health care in female prisons, including mental health research with women who engage in “self-injurious behavior” [1]. In 2014, the Office of the Correctional Investigator (the review body that acts as the ombudsperson for federal offenders) called for more research into the causes of suicide among prisoners [2]. Thus, suicide prevention in prisons, as an area of research, seems to be of high priority. Moreover, when asked about their greatest areas of concern with the correctional system, 10.4% of Canadian prisoners cited “health care”, second only to “conditions of confinement” [3]. Several diseases, in fact, affect prisoners at rates higher than in the general population, e.g., Hepatitis C, cardiovascular disease, mental illness, and substance use disorders, suggesting that how best to treat these diseases in prisons requires greater research, too.

Recent reviews of the health of people incarcerated in Canada and a variety of other countries (e.g., the United States, Australia, Iran) illustrate a disturbing lack of research, especially evidence on interventions to improve health among these populations [4, 5]. There is an urgent need for more health and social science research to improve our understanding of the health consequences of incarceration in Canadian prisons and to ensure that prisoners are afforded treatment that is consistent with Canadian rights and values. Despite the clear need for greater research, for researchers outside the correctional system, it is challenging to gain access to information about what health research is conducted with provincial and federal prisoners in Canada and the amount and types of resources dedicated to this research.

Despite the real benefits that research has made possible for prisoners, e.g., research demonstrating the benefits of needle exchange programs in reducing transmission of HIV/AIDS in prisons, [6] prisoners may remain prone to abuse during the conduct of health research given the inherently restrictive nature of prisons and the power imbalance between prisoners and officials. The history of research with prisoners is punctuated with such examples of ethically disturbing incidents [7]. For example, the syphilis studies in Guatemala, sponsored by the U.S. National Institutes of Health during the late 1940s, included intentionally infecting prisoners with syphilis, without their consent, to test antibiotics [8]. During the 1950–70s, inmates at the Holmesburg Prison in Pennsylvania were exposed to pathogens during the course of research without their consent in a series of dermatological experiments sponsored by private companies [9]. In Canada, little is available on historical accounts of experimental research with prisoners. Two academic papers provide accounts of pharmacological research with prisoners, notably LSD, sensory deprivation and behavioural experiments between 1955 and 1975 [10, 11]. Dorothy Proctor, a teenage inmate, was part of an experiment with other women incarcerated in the Kingston penitentiary who were administered electroshock therapy, sensory deprivation, and LSD.

Unlike the United States, where the protection of human subjects in research is governed by federal regulations, including specific provisions regarding research with prisoners, [12] in Canada the *Tri-Council Policy Statement 2* (TCPS) and other research ethics guidelines (e.g., those of Correctional Services Canada– CSC- or Ontario’s Ministry of Community Safety and Correctional Services - MCSCS) provides only limited guidance on research with prisoners. For example, the Commissioner’s Directive on research by the CSC states that research with prisoners must contribute to the mission of the CSC, comply with the *Corrections and Conditional Release Act 1992*, and comply with the TCPS [13]. In Ontario, the MCSCS research guidelines state that researchers must submit a proposal to the Correctional Services Research Committee that will be evaluated on the basis of such things as the “… potential value of the project to Correctional Services and the Ministry” and “imposition on the host site(s) in terms of disruption to Correctional Services”, but provide very little tangible guidance for researchers [14]. For example, while conducting a study designed to understand the health-related challenges of imprisoned women returning to their communities in Canada, one of us (Matheson) identified barriers to participant recruitment related to restrictions on the use of incentives for research participants in prisons and under community supervision [15]. Matheson and colleagues found no national standards for how researchers should design and use incentives for prisoners to participate in research, and found that policies and practices vary across Canada. This lack of research ethics guidance, federally and provincially, creates troubling uncertainties for researchers.

The purpose of this paper is to encourage a national conversation about what it means to conduct ethically sound health research with prisoners given the current realities of the Canadian system – though we acknowledge at the outset that the arguments likely extend beyond health into other realms of social science research. We begin with a review of the current TCPS guidance on research with prisoners in order to propose concrete steps to further the discussion and improve current guidance on this topic. (Although the TCPS only applies to researchers who have obtained funding via one of Canada’s three federal funding agencies, it is likely that many scholars who would like to conduct research with prisoners will have funding from one of these agencies at some point in time or reside in universities or institutions whose research ethics boards (REB) require compliance with the TCPS regardless of the source of funding.) In particular, we argue that meaningful dialogue and engagement must occur with various stakeholders, including prisoners, staff of correctional services across Canada, and health researchers themselves in an inclusive and respectful manner.

## Main text

### Health of Canadian prisoners and implications for research ethics

There were approximately 144,000 adult offenders in custody or community programs per day in Canada during 2014 and 2015, the vast majority of whom were under community supervision (82%). During that same time period, women accounted for 15% of adult admissions in provincial and territorial correctional services [16].

Two major trends in prison populations present particular challenges for research ethics in Canada. First, Indigenous peoples, which include First Nations, Inuit, and Métis communities with diverse cultures and languages, account for 25% of prisoners in provincial or territorial custody and 22% in federal custody, yet they represent only 3% of Canada’s overall population [16]. There are even greater challenges with respect to Indigenous women who represent 38% of the sentenced population compared to 3.8% of the Canadian population [16]. The cultural atrocities experienced by Indigenous women in the form of “historic trauma and inter-generational grief” figures prominently in the incarceration, physical health, mental health, and substance use disorders of some prisoners [17]. Given the likelihood that health research with Canadian prisoners would include Indigenous peoples, their values and belief systems must be accounted for by researchers and REBs. A starting point would be Chapter 9 of the TCPS, which focuses on engagement with First Nations, Inuit, and Métis communities and the recognition that even if someone is separated from their community, e.g., while in prison, Indigenous persons still have beliefs, practices, and processes of governance that must be respected during participation in health research. The onus on upholding and promoting the values of Indigenous people in prisons who engage in health research lies not only with the researchers themselves, but also with correctional services and all other institutional actors. In particular, CSC has a document titled *Strategic Plan for Aboriginal Corrections* informed by Elders, Indigenous Liaison Officers, community representatives and Indigenous organizations. As such, CSC and its Indigenous partners are well-positioned to inform ethical standards and research practice for Indigenous inmates, [18] although there are documented instances where the power of Elders to inform and influence practice in correctional facilities has been of limited success [19]. Researchers must be given the space and resources (e.g., the ability to use sacred tobacco and sweet grass, considered a sacred plant in many Indigenous communities, as a gift and sign of respect) to uphold Indigenous beliefs and values in pursuit of sound and practical health research. Researchers, correctional services, and Indigenous peoples also have an important opportunity and responsibility to consider how best to situate health research within the context of the Truth and Reconciliation Commission of Canada’s *Call to Action* [20].

Second, in 2008, 13% of male inmates and 29% of female inmates presented with mental illnesses at admission to prisons, and 45% of males and 69% of female inmates required mental health interventions while in custody [21]. The deleterious psychiatric effects of prisons themselves are compounded by the increasing rates of segregation for prisoners with mental illnesses, including those at risk for suicide (an increase of 6.4% over the last five years) [3]. Thus, psychiatric and mental health services research may be particularly valuable in prisons. However, researchers will need to address the potential stigma of participation in mental health services research for inmates. Researchers must be aware of the many consequences that stigma might have for prisoners who engage in mental health research. Although important strides have been taken in recent years to combat stigma associated with psychiatric diagnoses, [22] these illnesses are still thought of, by the general population and often by patients themselves, as a sign of weakness [23]. For example, participating in psychiatric and mental health services research may further stigmatize those participants enrolled as ‘weak’ in the eyes of correctional staff, prisoners, or the participants themselves, which may make them more vulnerable to physical or sexual assault, given the aggressive nature of many prisons. REBs and correctional oversight committees ought to be sensitive to such challenges and be willing to work closely with researchers working in correctional institutions to find ethical and practical solutions, but their effectiveness is currently limited by inadequate guidance.

### TCPS and research with prisoners

The TCPS provides three ethics principles to guide research in Canada: first, *respect for persons* acknowledges the intrinsic value of all humans and is manifested in practice by protecting the autonomy of participants through processes like informed consent. Second, researchers ought to have a *concern for the welfare* of participants, which means seeking concrete ways to promote the physical and mental well-being of participants and protect them from harm. Third, the principle of *justice* requires that participants are treated fairly (i.e., treating all persons with equal respect) and equitably (i.e., a fair distribution of benefits and burdens of research) [24].

Two distinct challenges for research with prisoners are raised explicitly in the TCPS: the implications of power differentials and structural obstacles to voluntary participation in research; and challenges in protecting the privacy of prisoners and the confidentiality of their personal information. In this section, we elaborate on how these challenges might present themselves in practice.

#### Power differentials and structural obstacles to voluntariness

The TCPS explicitly acknowledges that certain populations, including prisoners, may be vulnerable to abuse by researchers. The term “vulnerable”, which is used but not defined in the TCPS, is controversial and open to interpretation. In the case of prisoners, particularly those with mental illnesses, the causes of vulnerability may stem from reduced access to “social goods, such as rights, opportunities and power” [25]. Power differentials exist between prisoners and those who work in the correctional system because the purpose of the correctional system is, in part, to limit a person’s freedoms. However, power differentials also exist because of social challenges that could potentially impede rehabilitation within the correctional system. For example, the lack of available community mental health resources may be a reason why persons with psychiatric illnesses are over-represented in the criminal system. Their poor health is exacerbated by a lack of mental health resources within prisons themselves. Researchers must navigate such existing dynamics and remain vigilant about the potential to abuse their own power over prisoners.

The existing power differentials then raise difficult and unique questions about the voluntariness of prisoners’ participation in research [26]. According to the TCPS, undue influence is “the impact of an unequal power relationship on the voluntariness of consent” and occurs “when prospective participants are recruited by individuals in a position of authority” [27]. For example, a prisoner may feel unable to decline participation or may agree to participate in research due to boredom or because they believe doing so will have positive future consequences while in prison.

A researcher’s concern for the welfare of prisoners as participants and their respect for prisoners as persons might be severely limited in practical terms, if background supports are lacking (e.g., lack of routine medical care, staff knowledge of the importance of research which may affect cooperation, or crisis assistance if participants decompensate during interviews). For example, prisoners may think that access to clinical research is their only chance for medical care and therefore may take on greater risks than they would under different circumstances. Researchers must be conscious of how the current correctional system may negatively affect prisoners and challenge their own ability to conduct ethical and scientifically sound health research. For example, a lack of access to prisoners in maximum security or those in segregation could skew potentially important research about the experiences of Canadian prisoners, their health, and their experiences with the healthcare system while in federal or provincial/territorial custody.

However, taking respect for persons seriously would also entail allowing prisoners to make choices regarding what they believe is acceptable risk in research, avoid being over-protective of prisoners, and to give various prison populations the opportunity to benefit from research. How best to respect the prisoner as a person, while not unduly influencing him or her to participate in research, is difficult to navigate in practice. The case of incentives with prisoners, as described above, illustrates some of the complexity in upholding the principle of respect for persons. An incentive is “anything offered to participants, monetary or otherwise, for participation in research” [28]. According to many correctional jurisdictions in Canada such as the CSC, “there can be no rewards or incentives for inmates participating in research projects” [29]. This varies by jurisdiction with some allowing incentives on a case by case basis and others allowing incentives for people under community supervision, but not for those in custody. Matheson and colleagues argue that not offering incentives may discriminate between prisoners and other non-prisoner research participants who do receive incentives for the same or similar studies [15].

#### Challenges in protecting privacy and confidentiality

Researchers also face challenges about how to best maintain the privacy and confidentiality of prisoners engaged in research, e.g., in the face of subpoena or seizure of research data. For example, there are several documented cases of breaches of prisoners’ privacy in the course of their participation in research in other countries [30]. Health researchers may be pressured by members of the correctional system or police to provide them with information on prisoners [31]. For example, prison administrators or police may confiscate interview recordings or transcripts about prisoners’ experiences with health services while in prisons. However, a recent ruling by the Quebec Superior Court protected the rights of researchers from the University of Ottawa to maintain the confidentiality of the video interviews they conducted with Luka Magnotta prior to his arrest and conviction for first degree murder of Jun Lin [32]. This ruling would suggest that Canadian courts may grant some protection of privacy and confidentiality to participants faced with criminal charges, though the legal boundaries remain murky and it remains unclear whether the Quebec ruling will sway courts in other Canadian jurisdictions. The TCPS advises that all measures taken by researchers to protect privacy and confidentiality, and any potential limitations of these measures, must be described to prospective participants during the informed consent process, including the potential limitations of researchers to protect their participants and data in light of pressure from police or other authorities.

## Conclusion

It is unclear what research ethics policies and procedures must be followed to ensure the well-being of Canadian prisoners who enroll in health research. The TCPS identifies some relevant challenges and considerations, but provides few concrete recommendations. Moreover, the recent TCPS interpretations by the Panel on Research Ethics, which are intended to clarify ambiguities in the TCPS, provide no specific recommendations about how to interpret the guidelines in relation to research with prisoners since the Panel has received no questions pertaining to this matter to date [33]. The CSC requires that researchers conducting studies with prisoners should follow the TCPS, while MCSCS requires hospital or university REB approval before a research contract is ratified. With so little explicit guidance at the disposal of REBs it is not clear that these procedures provide adequate and consistent protections for participants. Moreover, there are other ethical issues related to research ethics and prisoners that are not addressed in the TCPS and that require further attention than we have provided in this paper, including, how best to incorporate the Truth and Reconciliation Commission’s *Calls to Action* into correctional services in Canada; challenges related to health research and the overrepresentation of minority races and ethnicities in Canadian prisons, e.g., Black Canadians; [34] and challenges in implementing and evaluating harm reduction strategies for substance use disorders [35].

The uniformity of research ethics practices in prisons across Canada remains unclear. Differences in the use of incentives have already been noted by Matheson and colleagues, and it is likely that other differences in important research ethics practices exist as well, for example in research ethics review processes. Research conducted through universities, and affiliated research institutes, will be vetted by REBs, but it is unclear the extent to which internal research commissioned and conducted internally by correctional authorities across Canada receives formal research ethics review and oversight. Greater transparency of these processes would enhance the trustworthiness and legitimacy of the research ethics functions of the correctional authorities.

In order to improve the transparency and processes associated with health research and research ethics in Canadian prisons, there ought to be disclosures of reasons for why decisions are made, to the fullest extent possible, particularly for refusals of health studies. We believe that stakeholder and community engagement are key because no one group can solve these ethical challenges on their own given their inherent complexity. Given this complexity, stakeholder and community engagement and dialogue should precede and inform a meeting or series of meetings with key actors to establish a clearer set of research ethics standards for Canadian prisons, and identify any other gaps in existing policies or legislation. In particular, there is a need for consultation and dialogue with prisoners, CSC staff (and other correctional authorities), and health researchers themselves on what it means to conduct ethical research in Canadian prisons. In their role as gatekeepers for external research with prisoners, correctional service staff are able to ensure that potential participants are not over-burdened by research participation. For example, special populations such as women and Indigenous peoples are of great interest to external health researchers, but this interest could quickly result in participant fatigue without the formal monitoring provided by these departments. Partnerships between external researchers and correctional staff are valuable since staff members are highly knowledgeable about the daily routine of prison life, which can inform the research design (e.g., recruitment strategies). However, this gatekeeper function must be guided by rules and reasons that are transparent to all stakeholders. It is imperative to create mechanisms to accent the positive and protective aspects of the gatekeeping function, while minimizing potential negative aspects as discussed above.

As importantly, the viewpoints of prisoners on their own health and what constitutes sound ethical research cannot be overlooked. It must be presumed that prisoners understand their own interests better than correctional staff or health researchers who may have never experienced the perils of prison life. Dialogue with prisoners and CSC and provincial services such as MCSCS staff must also include the voices of the health researchers themselves, who must be given the space to express their concerns related to the conduct of ethically sound research. Building an open dialogue among researchers, correctional staff, and prisoners will not occur quickly, but serious efforts to create the appropriate spaces and environment for the necessary dialogue would be a critical first step to developing meaningful ethical guidelines for research with prisoners. REBs and other relevant research oversight committees should also be involved in this dialogue, since they are best positioned to identify issues and trends across a host of research projects and communicate best-practices they may identify to investigators in their institutions.

Although the federal approach to research ethics policy in Canada has been to try to capture all research involving human beings under a single, over-arching policy, the current circumstances in Canadian prisons may warrant some additional protections for prisoners. Therefore, at the very least, we believe that the time has come for national research ethics organizations to examine the sufficiency of the existing research ethics guidance and policy landscape. A conference convened by the Interagency Advisory Panel on Research Ethics (PRE or the Panel) representing the three federal research funding agencies, the Secretariat on Responsible Conduct of Research (SRCR or Secretariat), or the Canadian Association of Research Ethics Boards (CAREB) all would be ideal places to begin this conversation, along with the major federal funding agencies, which support the research in question—i.e., the Canadian Institutes for Health Research, the Social Science and Humanities Research Council, and to a lesser degree the National Sciences and Engineering Research Council. There ought to be a disciplinary association of those interested in health and social science research with prisoners to consolidate ideas and create guidelines for ethical research with Canadian prisoners. As a global leader in research ethics, Canada should do more to ensure that research with prisoners is uniformly conducted, at the very least, according to the core values of the TCPS.
